# Left Ventricular Assist Device Management in the Emergency Department

**DOI:** 10.5811/westjem.2018.5.37023

**Published:** 2018-07-26

**Authors:** Paul Trinquero, Andrew Pirotte, Lauren P. Gallagher, Kimberly M. Iwaki, Christopher Beach, Jane E. Wilcox

**Affiliations:** * Northwestern University, Feinberg School of Medicine, Department of Emergency Medicine, Chicago, Illinois; † University of Kansas School of Medicine, Department of Emergency Medicine, Kansas City, Kansas; ‡ St. Luke’s Hospital, Department of Emergency Medicine, New Bedford, Massachusetts; § Northwestern University Feinberg School of Medicine, Department of Medicine, Division of Cardiology, Chicago, Illinois

## Abstract

The prevalence of patients living with a left ventricular assist device (LVAD) is rapidly increasing due to improvements in pump technology, limiting the adverse event profile, and to expanding device indications. To date, over 22,000 patients have been implanted with LVADs either as destination therapy or as a bridge to transplant. It is critical for emergency physicians to be knowledgeable of current ventricular assist devices (VAD), and to be able to troubleshoot associated complications and optimally treat patients with emergent pathology. Special consideration must be taken when managing patients with VADs including device inspection, alarm interpretation, and blood pressure measurement. The emergency physician should be prepared to evaluate these patients for cerebral vascular accidents, gastrointestinal bleeds, pump failure or thrombosis, right ventricular failure, and VAD driveline infections. Early communication with the VAD team and appropriate consultants is essential for emergent care for patients with VADs.

## INTRODUCTION

Heart failure (HF) produces a significant disease burden in the United States, with over 5.1 million Americans suffering from HF and over $32 billion expended annually. Although survival from HF has improved, the mortality rate at five years is 50%.^1^,^2^ Ventricular assist devices (VAD) have improved survival in patients with advanced HF.^3^ Over 22,000 patients with advanced HF have received VADs in the last decade, and implantation rates are expected to increase with newer generation devices.^3^ VADs may be used as “destination” (e.g. permanent) therapy or as a “bridge to transplant” (BTT). Patients implanted as “destination therapy” will remain on the VAD for the rest of their lives. BTT patients will remain on their VADs until they undergo heart transplantation.

Many patients with VADs are well informed about their devices and possess adequate VAD self-management skills. This includes contact information for VAD centers and instructions on obtaining assistance when needed. In addition, VAD patients are generally accompanied by a VAD-trained caregiver (e.g. family member). Despite precautionary measures, including close outpatient follow-up and detailed instructions on the device, the incidence of VAD patients presenting to the emergency department (ED) will likely increase due to rising rates of VAD implantation. Thus, emergency physicians must be proficient with the diagnoses and treatment of VAD-related emergencies and general management of VAD patients. Optimal treatment requires understanding of the associated anatomy and changes in cardiovascular physiology associated with VADs, and knowledge of the device itself. This article provides emergency physicians with an overview of the current U.S. Food and Drug Administration (FDA)-approved assist devices and provides a framework for patient assessment, including common VAD-related complications, device troubleshooting, and the management of the unstable VAD patient.

### Overview of Current Left Ventricular Assist Devices (LVAD) and VAD Components

LVADs are surgically implanted into the apex of the left ventricle of the heart (inflow cannula) and are connected to the aorta via an outflow cannula providing circulatory support to the patient. A driveline passes from the device through the skin, connecting to a system controller that in turn is connected to external power. While the first generation VADs were pulsatile, all current devices are continuous flow, which have improved survival and lowered rates of device failure.^4^ This paper will focus on the second- and third-generation continuous-flow VADs currently approved by the FDA, as very few patients still have first- generation VADs.

The HeartMate II™ (HMII), the HeartMate III™ (HMIII), and the HeartWare® (also called HVAD) are the three FDA-approved assist devices. The characteristics of the various pumps are highlighted in the table. In the case of an obtunded or altered patient, this table provides a reference to distinguish devices and information to relay over the phone to the VAD team or implanting hospital.

Notable VAD components include the driveline, controller, and battery pack.^5^ The driveline is tunneled from the device to the skin and exits through the abdominal wall. It forms the connection between the surgically implanted VAD, which is located in the thoracic cavity (HVAD, HMIII) or intra-abdominal cavity (HMII), and the external controller. The controller serves to convey pump function parameters and alarms as well as provide a medium to adjust the device settings. Finally, an external, replaceable, and rechargeable battery pack powers the device.

### Initial Approach and Emergency Department Management

Evaluation, management, and troubleshooting for patients with a VAD represent a unique clinical challenge as the presence of a mechanical support device changes native cardiovascular physiology. The evaluation of the stable VAD patient is similar to other patients, and should appropriately address the chief complaint. Because seemingly minor ailments can mask more significant pathology, the VAD team/coordinator should always be contacted. This will mobilize appropriate resources and facilitate communication. In hospitals with a cardiothoracic intensive care unit or VAD unit, evaluation of VAD patients (particularly vital signs) can often be facilitated through the services of the on-call VAD nurse/tech/physician. Given the complexity and increase in utilization of durable mechanical support devices, it is appropriate for all EDs and urgent care facilities to have a written protocol in place to provide optimal care for patients with VADs.

**Figure 1:**
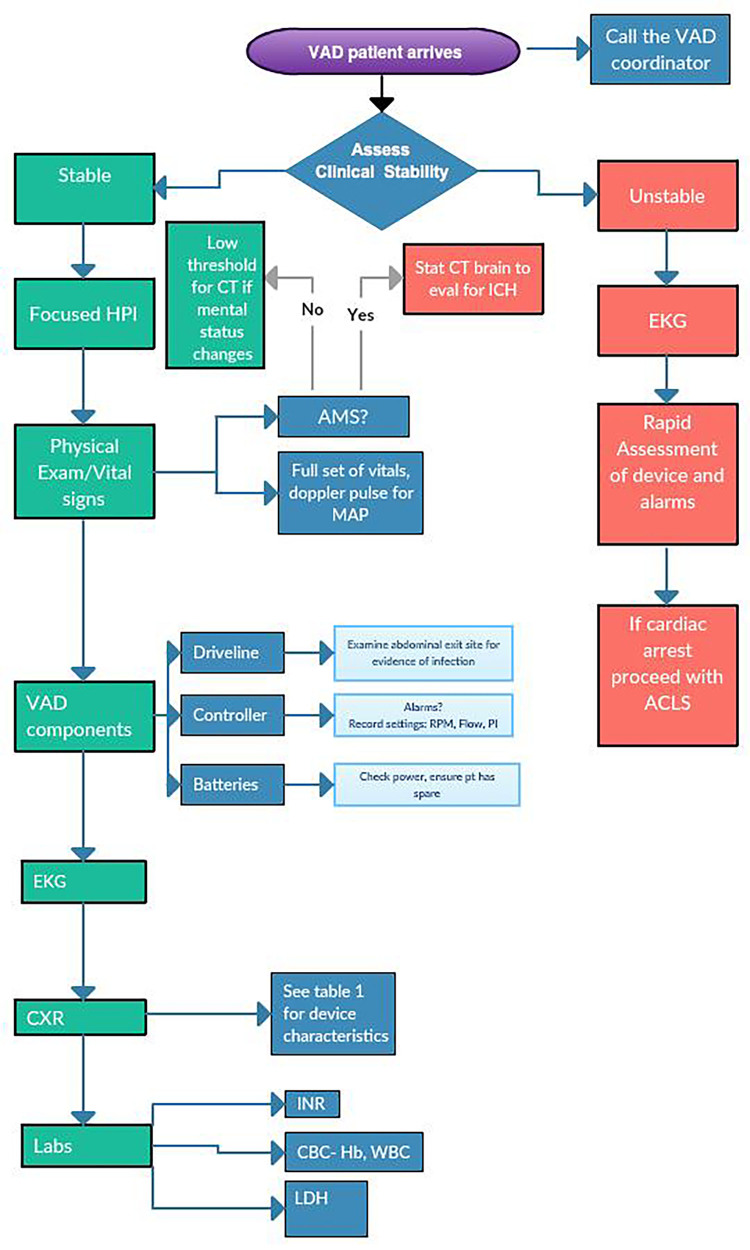
Emergency department approach to VAD patient. *VAD*, ventricular assist device; *HPI*, history of present illness; *CT*, computed tomography; *ICH*, intracranial hemorrhage; *EKG*, electrocardiography; *AMS*, altered mental status; *MAP*, mean arterial pressure; *RPM*, revolutions per minute; *PI*, pulsatility index; *PT*, patient; *ACLS*, advanced cardiovascular life support; *CXR*, chest x-ray; *CBC*, complete blood count; *Hb*, hemoglobin; *WBC*, white blood cell count; *LDH*, lactic acid dehydrogenase.

**Figure 2:**
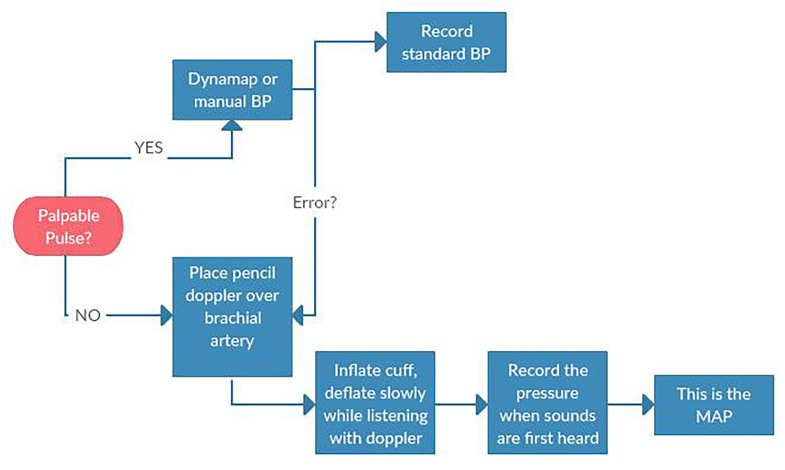
Obtaining a blood pressure (BP) for patient with Ventricular Assist Device. *MAP*, mean arterial pressure.

Primary ED evaluation begins with a full history, physical, and evaluation of the device (Figure 1). Heart rate is variable depending on the patient’s intrinsic rate and rhythm. Many patients with VADs also have cardiac pacemakers or implantable cardioverter defibrillators (ICD) in place. The continuous-flow VAD device does not generate a pulse, but patients may have enough residual or recovered ventricular function to mount intrinsic pulsatile flow. Because the degree of pulsatility is variable among VAD patients, a standard approach to measuring a blood pressure is recommended (Figure 2). Given the continuous-flow pump characteristics, measuring the mean arterial pressure (MAP) is the most reliable measure of perfusion pressure and is standard of care for VAD patients. First, palpate the radial artery. If a pulse is present and consistent, obtain a blood pressure using a standard sphygmomanometer. If unable to obtain a blood pressure reading or if there is no pulse, use the Doppler method to obtain the MAP: Place a pencil Doppler probe over the brachial (or radial) artery and inflate a blood pressure cuff 30 millimeters of mercury (mmHg) past when the arterial pulse is no longer detected by Doppler. Slowly deflate the cuff until arterial flow is once again audible. The corresponding pressure is the MAP. If unable to reliably measure MAP using the Doppler method, consider an arterial line to evaluate perfusion. Due to continuous flow, the arterial line waveform will often remain flat or have minimal pulse pressure.

Continuous-flow devices are very sensitive to afterload. Higher mean arterial blood pressures lead to increased afterload on the device and may lead to decreased pump flow. Clinically, this may manifest as worsening symptoms of HF. Increased afterload can also lead to subendocardial ischemia, which may potentiate ventricular arrhythmias. Adequate MAP control is essential in VAD patients; current guidelines recommend a target MAP <80 mmHg as long as symptomatic hypotension can be avoided.^4^ The Interagency Registry for Mechanically Assisted Circulatory Support has defined a hypertension adverse event as MAP >110 mmHg for continuous-flow pumps.^6^ Angiotensin-converting-enzyme inhibitors and beta blockers are the preferred agents for outpatient management of blood pressure.^2^ Oral hydralazine is often a preferred antihypertensive agent for reducing blood pressure in the ED.

### Physical Exam Including VAD Components

Evaluation of a stable VAD patient should include a focused physical exam and inspection of the major device components. Cardiac auscultation facilitates rapid evaluation of the device; in a properly functioning VAD, a “whirring” sound should be heard. By definition, patients with VADs should be relatively free of signs and symptoms of HF due to the presence of the mechanical support device. Thus, any signs of volume overload (e.g., elevated jugular venous pressure, presence of ascites or peripheral edema) may be indicative of subacute or chronic right ventricular failure, while shortness of breath, pulmonary edema, or hypotension are often present with acute device malfunction (e.g., pump thrombosis, cannula obstruction).

Distal perfusion should be assessed via capillary refill or simply by palpating the extremities. Because of an increased propensity for bleeding, the VAD patient should be evaluated for focal neurologic deficits, change in mental status, or presence of headache with a stat non-contrast computed tomography (CT) of the brain to rule out intracranial hemorrhage.^7^

The VAD driveline exit site will be covered with a sterile dressing and should be inspected carefully in a sterile fashion (mask, gloves) for any evidence of infection. The controller should be inspected and current settings and pump parameters recorded, including any alarms. Finally, ensure that the patient has brought along his or her back-up batteries and controller.

### Relevant Studies and Workup

Initial workup in a VAD patient centers on the chief complaint similarly to non-VAD patients with significant cardiac disease. Electrocardiogram (ECG) findings in VAD patients may be nonspecific, but in addition to stigmata of end-stage heart failure they tend to include low limb lead voltage, ubiquitous electrical artifact, and QRS splintering.^8^ Although the VAD performs the primary left ventricular pumping function, the native heart still contributes to cardiac output. The right ventricle must provide adequate preload to the left ventricle and subsequently fill the LVAD. Accordingly, although some VAD patients may have a higher tolerance for ventricular arrhythmias, if the patient becomes unstable or symptomatic, termination of the ventricular arrhythmia is paramount. In most cases, this will require electrical cardioversion, although intravenous (IV) doses of antiarrhythmic medications such as amiodarone can be given simultaneously and may reduce recurrence.^9^,^10^ One important etiology of ventricular arrhythmia in a VAD patient is a suction event, which occurs when the inflow cannula contacts and stimulates the ventricular septum.^11^,^12^ This occurs as the result of decreased left ventricular (LV) filling (potentially from hypovolemia), myocardial recovery, or excessive pump speed. Treatment of suction events includes a fluid challenge and/or adjusting the device speed in conjunction with the VAD team.

The chest radiograph (CXR) is an important diagnostic tool for VAD patients. Direct visualization of VAD positioning as well as presence/absence of ICD aids the emergency physician in baseline evaluation. CXR can also help to identify the particular device if it is not otherwise apparent (Table).

Laboratory workup is vital in the evaluation of VAD patients. All patients with VADs are anti-coagulated with vitamin K antagonists (e.g., warfarin) with an international normalized ratio (INR) goal of 2.0–3.0 unless contraindicated.^7^ Troponin (troponin T, hsTnI), creatine kinase-MB, and myoglobin may be useful in evaluating VAD patients with chest pain or ECG changes. Elevated brain natriuretic peptide (BNP) (or NT-proBNP) may help identify right heart failure, or pump thrombosis/malfunction. As BNP is primarily an atrial responsive agent, it remains a useful marker for identifying volume overload in VAD patients and can guide therapy (e.g. diuresis). Finally, lactic acid dehydrogenase (LDH) is useful in screening for evidence of hemolysis. LDH levels 2.5 times upper limit of normal are suggestive of pump thrombosis in the appropriate clinical setting.^7^

### Specific Complications in VAD Patients

Complications unique to VAD patients can be classified as “VAD-specific” and “VAD-associated.” VAD-specific complications include 1) pump failure/malfunction, and 2) pump thrombus. These will be discussed in detail in the next section. VAD-associated complications include the following: 1) gastrointestinal (GI) bleeding, specifically related to the presence of arteriovenous malformations (AVM); 2) cerebrovascular accidents (CVA), either embolic or hemorrhagic in etiology; 3) VAD driveline infections, which may be localized to the percutaneous exit site or deeper within the pump or pump pocket;^13^ and additionally, 4) right ventricular (RV) failure occurs in 15–20% of VAD patients and can lead to persistent HF symptoms and/or pump dysfunction (e.g. low flows).^14^

GI bleeding in VAD patients is multifactorial. Patients are maintained on lifelong therapeutic anticoagulation. Additionally, continuous-flow VAD patients will usually develop acquired von Willebrand factor (vWF) disease.^14^,^15^ The relatively higher shear stress brought on by non-physiologic circulation distorts the vWF multimers and leads to increased systemic cleavage and subsequent deficiency.^15^ Furthermore, VAD patients are susceptible to GI vascular malformations secondary to the decreased pulse pressure from continuous flow. A retrospective analysis of patients implanted with a HMII device found that 43% had a major bleeding episode requiring blood transfusion, the majority of which were localized to the GI tract.^14^ Management of GI bleeding often requires examination via endoscopy and colonoscopy for source control of AVM lesions. Blood transfusion should not be reflexive for the stable patient with GI bleeding, especially in BTT patients, as blood products are sensitizing and may reduce the chance of successful heart transplantation. In addition, robust transfusion of blood products will increase afterload and may lead to HF exacerbation. However, for larger GI bleeds or bleeding resulting in hemodynamic instability, blood transfusion is crucial. Because blood transfusion may lead to an increase in circulating antibodies and make it more difficult to find a donor match, transfuse with leukoreduced and irradiated blood products if available to decrease sensitization.^16^ Multidisciplinary consultation with VAD and transplant teams is essential in the management of GI bleeding.

CVA, either embolic or hemorrhagic, is often a devastating VAD-associated complication. In addition to the pro-thrombotic milieu of a failing heart, the implantation of a mechanical assist device creates a nidus for the formation of clots. Development of atrial fibrillation after VAD implantation is common, and increases risk of embolic CVA.^17^ Blood pressure control with a MAP <90, daily 81mg aspirin, and avoidance of supratherapeutic INR levels (>3.0) have shown to be effective at reducing stroke risk.^18^ Data from the ADVANCE trial estimates prevalence of ischemic CVA at 6.8% and hemorrhagic CVA at 8.4%.^14^,^18^ In the event of a CVA, early coordination with the VAD team and neurology/neurosurgery team is necessary to discuss reversal of anticoagulation and surgical options.

The driveline exit site provides a conduit for bacterial entry, making infection a relatively common VAD-associated complication affecting nearly 20% of patients within the first year of implantation.^19^ Infections may be superficial and localized to the percutaneous exit site or deeper within the pump pocket or pump itself.^13^ Blood cultures and driveline cultures should be obtained in any patient with suspected infection.^20^ Staphylococci are the most commonly isolated organism, but pseudomonas and other gram-negative bacteria are common culprits as well.^21^ Empiric antibiotics should be tailored to each individual patient. An abdominal CT is often helpful to evaluate for an associated fluid collection.^20^ In the event of systemic spread, management of sepsis mirrors that of non-VAD patients: aggressive fluid resuscitation; early delivery of antimicrobials; and central/arterial line placement as indicated. Central catheterization can be achieved from any of the routine sites. Unless patients have residual RV failure, the risk of “volume overloading” a VAD patient is generally low. Vasopressors may be appropriate after adequate volume resuscitation.

While the VAD provides circulatory support to the failing LV, RV failure is a common problem, occurring in 15–20% of VAD patients.^22^.^23^ Reduced preload to the LV leads to low VAD flows. “Low- flow alarms” on the VAD may be related to reduced preload from RV failure, but also may be secondary to hypovolemia or inflow cannula obstruction (less common, but a known complication). Laboratory markers of end organ dysfunction can aid in the diagnosis of RV failure. Elevated creatinine, liver transaminases, and the presence of lactic acidosis can indicate cardiogenic shock. If a shock state is suspected due to RV failure, inotropes (e.g. milrinone or dobutamine) should be used.

### VAD Device: Alarms and Troubleshooting

The parameters reported on the HMII and HMIII controller are speed, power, flow, and pulsatility index (PI). The HVAD controller reports speed and power only; waveforms are reported on the system monitor reflective of pulsatility. Speed is the only parameter that is set, in revolutions per minute. Power is measured in watts and is indicative of the work being done by the device. Flow is calculated based on the power and speed, and is a result of both the device speed and the pressure gradient between the inflow and outflow cannula. PI is related to flow through the device and can be thought of as the contribution of the native LV. As the native LV contracts, there is a pressure wave sent through the pump. The magnitude of this pressure pulse is measured by the device, averaged over time, and reported as the PI.

The HVAD device uses waveforms on the system monitor to provide an estimate of intrinsic LV function. A larger delta between the peak flow and trough flow represents greater contribution from the native LV. Clinical situations with less LV filling (e.g. hypovolemia) result in low PI. The PI may also be low if pump support is increased; blood is preferentially pulled into the device circuit and intrinsic LV volume is reduced. When troubleshooting the device, it is important to consider all of the parameters. For example, suction events often present with low flow, low power, and low speed because the device senses the event and responds by slowing down to allow for increased LV filling. In the setting of high flow and high power, pump thrombosis must be considered, especially when accompanied by signs and symptoms of HF.

The VAD controller will display, or alarm, in the setting of device malfunction or organic pathology interfering with device functioning. Immediate consultation with the VAD team is necessary. Make sure to properly identify the VAD type. Patients should know this information, which is found on the controller. Devices can also be identified by radiograph appearance as discussed above. An overview of each device and the corresponding alarm types is presented below. This is followed by a general outline of the approach to several common VAD alarms.

#### Heartmate II and III Alarms

The HM controller has two alarm icons: a battery and a heart. Most patients now have a pocket controller that has a user interface screen with further text information such as “Low Flow” or “Connect Driveline.” The battery alarm icon will flash either yellow or red to indicate the remaining charge. The yellow alarm indicates 15 minutes of remaining battery power. The red alarm indicates only five minutes remaining. The flashing red heart alarm indicates low flow or pump stoppage. This necessitates emergent discussion with the VAD team. In the setting of a red heart alarm ensure adequate IV access and maintain hemodynamic stability with inotropes as needed. See the discussion of low-flow alarms below for further detail.

#### HVAD Alarms

The HVAD controller has three levels of alarms, categorized by level of priority: low (solid yellow), medium (flashing yellow), and high (red). There are two parts to the display, an alarm and an action. In general, the alarms are intuitive. For example, the “Low Battery” or “Critical Battery” alarm is accompanied by an action such as “Replace Battery 1.” Even critical alarms such as “VAD Stopped” can have potentially easily reversible actions such as “Connect Driveline.” Several of the more common alarms are addressed separately below.

#### Controller Fault

VAD patients and their caregivers are instructed to carry a back-up controller in case of controller malfunction. This alarm necessitates immediate consultation with the VAD team. Controller exchanges should only be performed by a trained professional.

#### Electrical Fault

The driveline contains six separate wires to maintain pump function. There is a level of redundancy but fracture of these wires will cause an electrical fault alarm, and complete severance can result in pump malfunction.^24^ Consult with the VAD team immediately.

#### High Watts

Power spikes are often the result of pump thrombosis due to the increased energy requirement. The HMII and III devices - in the setting of obstructive thrombus - will display a flashing red alarm on the attached monitor; the HVAD monitor will read “Low flow – Call.”

#### Low Flow

**Figure 3:**
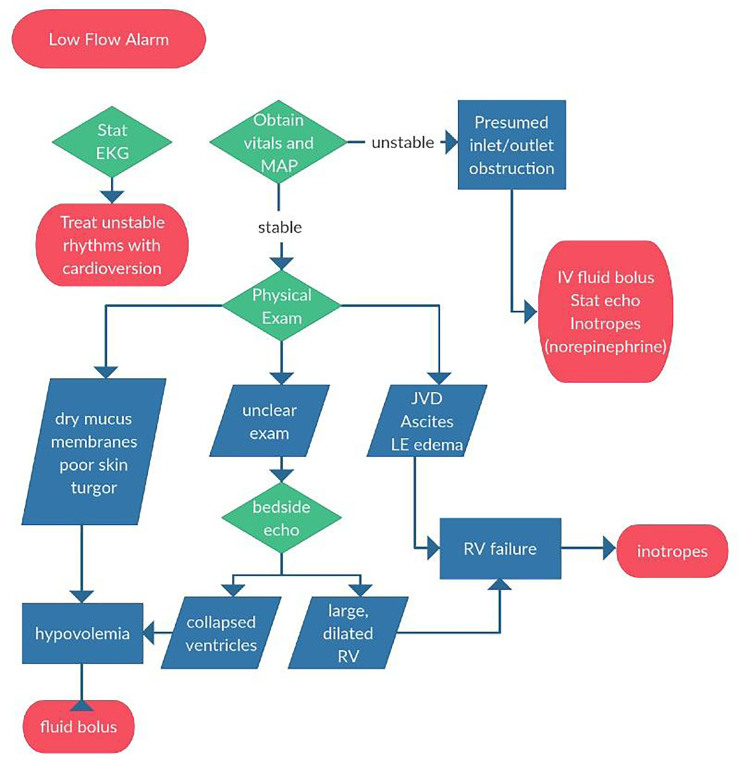
Approach to low flow alarms for patient with Ventricular Assist Device. *EKG*, electrocardiography; *MAP*, mean arterial pressure; *IV*, intravenous; *JVD*, jugular vein distention; *LE*, leg; *RV*, right ventricle.

Evaluation of a VAD patient with a low-flow alarm starts with an assessment of overall clinical stability (Figure 3). In a hemodynamically unstable VAD patient, a low-flow alarm should be treated as pump malfunction until proven otherwise. With a severe inlet cannula obstruction from thrombus flow through the VAD will be negligible and cardiac output is dependent on intrinsic LV function, which is likely insufficient to maintain adequate end organ perfusion. In this scenario, emergency physicians should treat the patient as you would any patient in cardiogenic shock.

Place large-bore IVs, obtain a stat echocardiogram (begin with a bedside point-of-care ultrasound if a formal study is not immediately available), and start inotropic support. Be sure to check a stat ECG, as ventricular dysrhythmias can precipitate acute right heart failure and subsequent shock. If the patient is stable, the provider should focus on differentiating other causes of low-flow alarms: hypovolemia and RV failure. If the etiology remains unclear after physical examination, a point-of-care ultrasound can be useful.

An inferior vena cava (IVC) that collapses on inspiration suggests inadequate pre-load and should be addressed with volume resuscitation.^25^ On the other hand, right heart failure or RV myocardial infarction may be detected by measuring the RV:LV ratio. On an apical four view, measure the widest diameter of each ventricle transversely from endocardium to endocardium. If the RV:LV ratio is greater than 0.6, this may indicate RV failure or RV strain.^25^ RV dysfunction can be suggested if the IVC decreases less than 50% with inspiration**.**^5^

Ultrasound can also be useful when troubleshooting other alarms. If both ventricles are large and dilated, this suggests pump failure, perhaps from thrombosis. Pump thrombosis is a true emergency and often requires surgical exchange of the device. Without pump exchange or transplant, pump thrombosis carries a 48% six-month mortality.^26^ Alternatively, a small LV could represent a suction event and can be addressed with a volume challenge and a discussion with the LVAD team about turning down the device speed.

### Management of the Unstable and Crashing Patient

When caring for an unresponsive or hemodynamically unstable VAD patient, one must emergently contact the VAD team while simultaneously stabilizing the patient. In a code, follow the conventional Advanced Cardiac Life Support algorithm including chest compressions, medications, and defibrillation as indicated. While there is a manufacturer warning regarding the risk of cannula dislodgment with manual chest compressions, the small body of available evidence suggests that this is rare.^27^ Withholding cardiopulmonary resuscitation in this scenario is universally fatal. Chest compressions should be performed on a pulseless VAD patient in an attempt to perfuse vital organs, while troubleshooting the device and contacting the VAD team.

Pulse checks should include brachial artery Doppler for MAP and review of the VAD monitor for signs of mechanical failure as discussed above. Auscultate the heart to listen for the “whirring” sound of the device. If you cannot hear the device functioning, troubleshoot the controller, ensure adequate power supply, and check all device connections. If the patient is hypotensive and has low VAD flows, consider a quick bedside ultrasound to evaluate for hypovolemia vs. RV failure. If the patient is hypotensive with elevated VAD flows, consider pump thrombosis, and also sepsis (extreme afterload reduction from vasodilation leads to higher VAD flows). If you suspect device malfunction, advanced therapies such as extracorporeal membrane oxygenation should be considered, especially in younger patients who are heart transplant candidates without significant comorbidities.

Evaluation of VAD patients can be daunting, but focused clinical priorities – taken in the context of the implanted device – facilitate rapid and appropriate management of both stable and critically ill VAD patients. Regular review of available support sources and quick access to reference material can greatly improve the care of VAD patients in the ED. Given the evolving technology and increasing prevalence of VADs, the ED community would benefit from both VAD-specific training programs in residency training and continuing medical education curricula.

## Figures and Tables

**Table:** U. S. Food and Drug Administration-approved assist devices.

	HVAD (Heartware®)	HeartMate II™	HeartMate III™
CXR Image	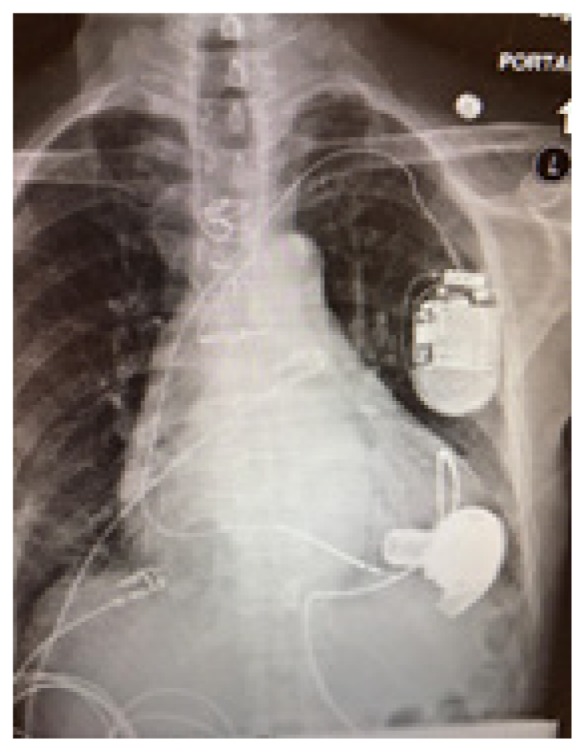	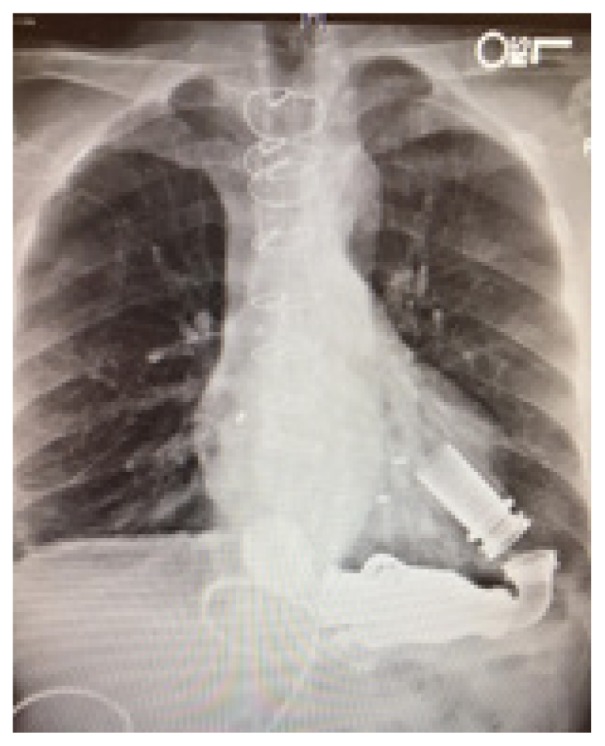	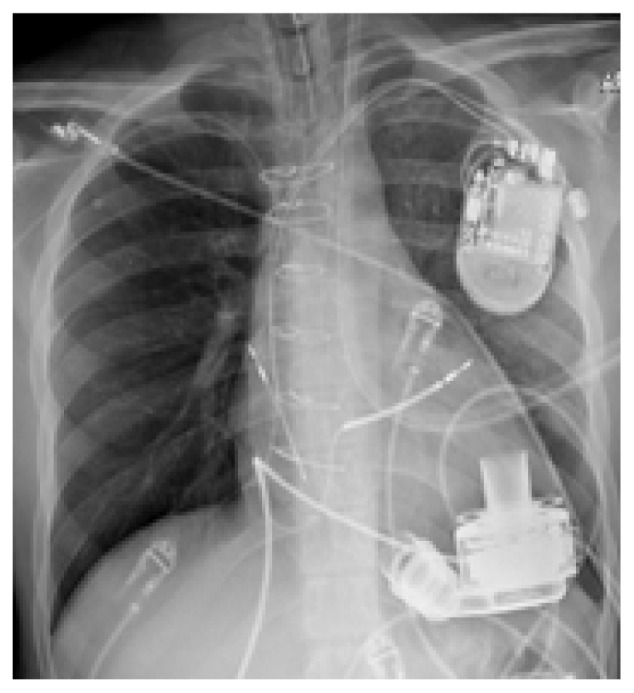
RPM	2500–3500	8800–10,000	4000–6000
Flow	4–6L/min	4–6L/min	4–6L/min
Controller	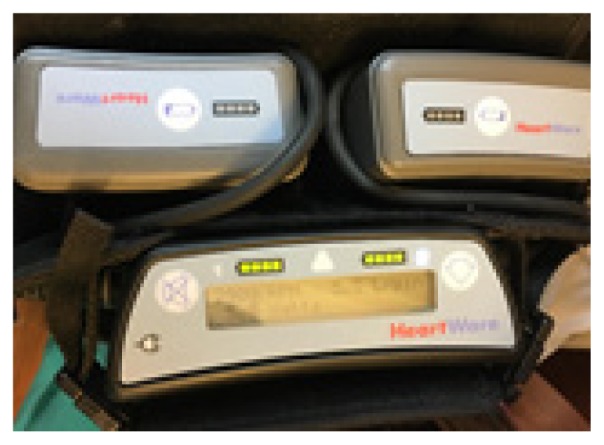	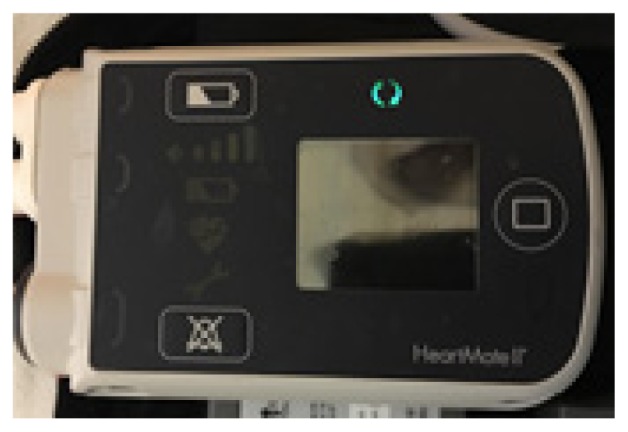	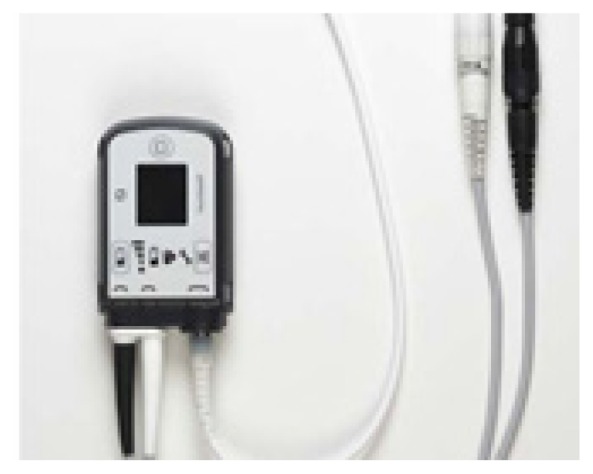

*CXR*, chest radiograph; *RPM*, revolutions per minute; *HAVD*, heart assist ventricular device; *L*, liter..
